# Identifying potential barriers and enablers to smoking abstinence after leaving a smokefree prison using the capabilities, opportunities, motivations -behaviour (COM-B) model: a qualitative interview study

**DOI:** 10.1186/s12889-025-23249-3

**Published:** 2025-06-05

**Authors:** Ashley Brown, Clair Woods-Brown, Kate Hunt

**Affiliations:** Institute for Social Marketing and Health, University of Stirling, Stirling, FK9 4LA UK

**Keywords:** Smokefree policy, Tobacco control, Prisons, Health, Qualitative

## Abstract

**Background:**

Smokefree prison policies reduce smoking-related harms among those living and working in prisons. Helping people released from smokefree prisons to remain abstinent post-release could deliver considerable additional benefits given high rates of relapse and the substantial burden of smoking on health. However, understanding of post-release smoking behaviour and the best ways to support people leaving prison who want to stop smoking for good is limited. No previous studies have explored how access to vapes in smokefree prisons may help or hinder people to remain tobacco-free post-release. The current study aimed to explore potential enablers and barriers to long-term tobacco abstinence after being released from a smokefree prison.

**Methods:**

Qualitative interviews conducted between 2022 and 2024 with people in prison (*n* = 27) and prison, health and third sector staff (*n* = 8) were transcribed and thematically analysed using the COM-B (‘capability’, ‘opportunity’, ‘motivation’ and ‘behaviour’) model of behaviour change to map facilitators and barriers to smoking abstinence post-release.

**Results:**

People leaving prison face substantial barriers to staying tobacco-free. Interactions between people rationalising smoking in the face of recognised harms (‘capability’), tobacco availability (post-release), pro-smoking norms, service limitations (‘opportunity’), competing needs and priorities and drug and alcohol use (‘motivation’) were identified as barriers. In contrast, desires to quit smoking and other ‘addictions’ which have caused substantial damage in people’s own and others’ lives, access to ‘smoking cessation’ services in prisons and positive social influence were identified as facilitators. Access to vapes in prison was perceived to have the potential to help or to hinder post-release smoking abstinence based on individual preferences and experiences.

**Conclusions:**

Reducing tobacco-related harms among people leaving prison and the communities they return to would help to reduce health inequalities and support other critical areas of public health and social justice work. Greater success requires overcoming considerable challenges, including those constraining prison and health services’ abilities to support positive behaviour change. Improved collaboration across services and expanded use of appropriately supported peer mentors and digital health interventions, may be both helpful and feasible in the current climate for reducing tobacco-related harms in a priority group.

**Supplementary Information:**

The online version contains supplementary material available at 10.1186/s12889-025-23249-3.

## Background

Smoking is a leading cause of preventable death and disability. Globally, ~ 8 million deaths, and 200 million disability-adjusted life-years were due to smoking in 2019 [1], and over 10% of deaths were attributable to smoking in Europe in 2021 [2]. Smoking prevalence and harms are higher among people experiencing multiple disadvantage [3], a group who are overrepresented among people in prison [4]. Estimates suggest around 15 million people who smoke are imprisoned annually worldwide, and that, where smoking in prison is allowed, smoking rates are up to 62 times higher in people imprisoned than in the general population [5]. High rates of smoking among people with experience of imprisonment contribute to health inequalities. A 2014 US study found that smoking attributable mortality and years of potential life lost rates were higher for those imprisoned than in the general population (360 vs. 248 and 5149 vs. 3501, per 100,000 respectively) [6]. Smoking is a risk factor for major non communicable diseases (NCDs) [7]. A study using administrative data for nearly 1.5 million people from eight countries found that 40% of deaths after release from prison were from NCDs, increasing with increasing age and time since release [8]. The ‘direct link’ [9] between NCD deaths (e.g. from cancers) among people with experience of imprisonment and smoking is evidenced in a 2007 Australian study which found that standardised mortality ratios for smoking-related cancers were significantly higher in men (1.7) and women (2.4) with experience of imprisonment, compared to the general population [10]. A 2009 US study found that higher all-cause cancer rates among people in prison were explained by smoking [11]. Smoking is also harmful to non-smokers exposed to second-hand smoke (SHS), resulting in 1.3 million deaths and 37 million disability-adjusted life years in 2019 [12]. In the UK, nearly one in three children in the general population had salivary cotinine levels indicating SHS exposure in 2018 [13]. Children affected by imprisonment may potentially be at greater risk for SHS-related illnesses, and for uptake of smoking themselves, given relatively high rates of parental smoking, and greater likelihood of indoor smoking occurring in disadvantaged households [14].

WHO Europe, among others [15], has described smoking among people with experience of imprisonment as a ‘*neglected public health issue’* [16]. Smoking receives significantly less attention in prisons than other substance use, despite very high levels of smoking and smoking-related harms among people entering prisons [17]. Data on implementation of tobacco control policies within prisons in Europe (and elsewhere) is limited [18]. Challenges with access to smoking cessation treatments in prisons has been highlighted as an issue in countries such as the US [19] and Australia [20]. Arguably, the area in which most progress has been documented is the implementation of comprehensive (i.e. indoor/outdoor) smokefree policies in prisons globally (e.g. New Zealand [21], US [22], Canada [23], Australia [4], Scotland [24], England and Wales [25]). If implemented successfully, such policies have been shown to (virtually) eliminate or substantially reduce active smoking and SHS exposures in prisons [26–28] and deliver corresponding health benefits. This includes, reductions in smoking-related deaths [6, 29], improvements in cardiovascular health [30] and reductions in prescribing for smoking-related illness [31]. There is limited evidence on smoking behaviours, and effective interventions to support long-term abstinence from tobacco use, following release from smokefree settings such as prisons [32, 33]. The relatively few studies which have been conducted in prisons in the US and Australia suggest that most people rapidly return to smoking post-release [32–34]. A 2024 scoping review [33] identified a small number of studies on factors influencing smoking behaviour after release from smokefree prisons and identified multiple drivers for smoking resumption. These included low or ambivalent motivation to quit smoking, having unrealistic beliefs about quitting smoking, experiencing stress, using drugs or alcohol, and returning to environments with a high prevalence of smoking. Similar factors were identified in an earlier review by Puljević and Segan [34]. While offering smoking cessation interventions either pre- and/or post-release from smokefree settings can increase rates of post-release smoking abstinence [32, 35], to our knowledge only one such intervention, reported in a 2013 US study, has demonstrated success in reducing relapse-to-smoking rates among people leaving smokefree prisons specifically [36]. Two more recent smoking cessation intervention studies conducted in smokefree prisons in Australia did *not* find evidence of an effect in reducing post-release smoking abstinence [37, 38]. However, these studies together with several other recent studies of smoking cessation interventions [39–42] delivered in prisons which *permit* smoking, demonstrate acceptability and feasibility of addressing tobacco use in prison settings. The paucity of smoking cessation/relapse prevention studies in prisons, particularly in relation to reducing post-release smoking abstinence, may partly reflect the significant and sometimes distinct challenges of delivering and evaluating healthcare initiatives in prisons [32], coupled with insufficient prioritisation of tobacco use in this population in the face of other significant operational, health and rehabilitative challenges.

The current qualitative interview study aims to contribute to the limited existing evidence [32] by using the COM-B model (see below) to explore potential enablers and barriers to smoking abstinence after leaving a smokefree prison. This evidence can help inform the development, adaption or refinement of interventions which are feasible and effective in preventing return to smoking among people who have left smokefree prisons. To our knowledge, only one previous study has qualitatively explored barriers/facilitators to post-release smoking abstinence [43]. No previous studies have explored in detail enablers and barriers to post-release smoking abstinence in prisons with a high prevalence of vaping. Understanding perspectives on whether and how vaping might help or a hinder long-term smoking abstinence post-release, alongside identification of other potential enablers/barriers, can help to inform development/adaption of interventions which are tailored to the needs of this population.

## Methods

This study presents findings from an analysis of qualitative interviews with people in prison, and interviews with relevant Scottish Prison Service (SPS), National Health Service (NHS) and third sector (non-government, non—profit organisation) staff. Interviews were conducted as part of a wider study which aimed to understand whether and how the public health benefits of smokefree prison policies in Scotland could be or had been maximised. Approvals for the work reported here were obtained from the NHS Research Ethics Committee (22/NS/0046), and SPS Research Access and Ethics Committee.

### Setting

We conducted the study in Scotland. All prisons in Scotland implemented a comprehensive smokefree policy on 30th November 2018. This policy prohibited people in prison from using tobacco (hereafter ‘smoking’) in all indoor and outdoor areas of the prison. Staff were already prohibited from smoking at work [44]. Rates of smoking in Scottish prisons were very high (estimated at 68%) in 2017, prior to the smokefree policy [45], reflecting high concentrations of smoking in disadvantaged communities in Scotland [46], and prison culture [9, 47]. In the lead up to prisons becoming smokefree, NHS ‘Quit Your Way’ services sought to provide timely access to free evidence-based treatments to people addicted to nicotine (pharmacological support [e.g. Nicotine Replacement Therapy] and/or behavioural support). Quit Your Way services continue to operate post-implementation [48]. However, prison healthcare services were badly disrupted by the COVID-19 pandemic [49], and continue to be under pressure e.g. due to workforce challenges [49], and a large and growing prison population [50].

Tobacco use is the main cause of cancer and preventable death in the UK [51]. While vaping is not risk-free [52], evidence to date suggests that (nicotine) vaping is less harmful to health than smoking [53] and can be effective for cessation [54]. Vapes started to be sold in prison shops (‘the canteen’) in Scotland in 2018, in anticipation of prisons going smokefree [55]. For a limited period in 2018, people in prison who smoked (tobacco) were eligible for a free vaping starter pack [56]. Six years on from implementation vaping rates in prisons are very high, even compared to those among people living in the most deprived neighbourhoods in Scotland, and vapes are a common way to administer illicit drugs in prisons [57]. Using figures collected between 2019 and 2024, vaping prevalence is thought to be 60% [58] to 76% [59] in prisons vs. ~ 12% in deprived communities [60]. In January 2020, guidance was launched for Quit Your Way practitioners working in prisons to support people who wanted to cut down and quit vaping, in recognition of need for this service [61]. However, the support did not have time to embed prior to the emergence of the COVID-19 pandemic.

### Sample and recruitment

The study began in 2022, but fieldwork was delayed/disrupted during the period in which COVID-19 restrictions and operational challenges were most affecting prisons. Interviews with people in prison were conducted in two Scottish prisons. For practical reasons, the research team asked NHS or SPS staff to help with recruiting people in prison and arranging interviews. Specifically, we sought help from staff to identify a varied sample of people who (a) smoked prior to imprisonment and were coming up for release and (b) who had recently returned to prison, had been imprisoned before and had ever smoked. Our rationale for interviewing people soon to be released from prison was to understand what continuities/changes in smoking behaviour were expected post-release and anticipated barriers/facilitators to smoking abstinence. We intended to also collect post-release data on smoking behaviours from some of the same participants. However, this was not feasible. We interviewed people who had recently returned to prison to understand past experiences of leaving a smokefree prison, what had helped or hindered long-term smoking abstinence, and any lessons learned. On the request of the prison service, we also asked to interview some people serving sentences of four years or more who were smoking prior to imprisonment, or prior to prisons going smokefree.

Recruitment proved particularly challenging in the post-COVID-19 period, given restrictions to movement within prisons and many other operational challenges at that time. Of the 27 people in prison interviewed, all were men. Twenty-six smoked tobacco prior to entering a smokefree prison/prisons going smokefree and one (ex-smoker) exclusively vaped before going to prison. Twenty-six were vaping while in smokefree prison; one was no longer vaping in prison. Ten were on remand, and 16 were sentenced (11 were serving sentences of less than 4 years, and 5 were serving sentences of 4 years or more; information for 1 person is missing). Most had recently entered prison or were approaching and contemplating release.

In addition, we invited prison, health and third sector staff working in relevant roles to participate in interviews. Staff were identified from the research team’s contacts with an understanding of those involved in providing services and support in this area, and suggestions from experts. Staff recruitment also proved challenging due to workforce pressures and other pressures in the prisons during this COVID-19 recovery period. Of the 8 staff interviewed, 3 worked for the SPS, 3 worked for the NHS and 2 worked for third sector organisations.

### Data collection

Interviews were conducted between 2022 and 2024 by experienced researchers (CWB, AB, KH and RO’D) using different topic guides for people in prison and staff (please see Additional File 1). The guides for people in prison covered: smoking and vaping histories, living in a smokefree prison (including vaping in smokefree prisons), preparing to leave a smokefree prison/past experiences of leaving a smokefree prison (if relevant), and views on how best to support people leaving prisons who want to remain smoking abstinent or make a home smokefree. Staff topic guides covered broadly similar topics. The topic guide for staff was updated in 2023 to explore priorities, opportunities and challenges for addressing tobacco use in prisons and wider society. Topic guides were used flexibly and participants were given the chance to raise any additional points they considered important. As far as possible, interviews with people in prison were conducted one-to-one in private rooms within the prisons without staff being present or able to overhear. Interviews with staff were conducted either in person or remotely, depending on participant preferences and logistics. Prior to starting interviews, the researchers provided participants with written and verbal information about the study and then recorded (verbal or written) informed consent from those who agreed to take part.

### Analysis and theoretical framework

Interviews were transcribed and de-identified prior to analysis. First, 17 interviews (completed to that point) were coded and summarised in Framework matrices (column = theme; row = interview) in NVivo 20 by AB and CWB. Completed matrices and interview extracts were used to identify key themes and sub-themes in the data. Subsequent discussions within the research team led to the identification of the COM-B model for behaviour change as a useful framework for further analysis relating to enablers and barriers to tobacco smoking abstinence post-release. According to the COM-B model, a behaviour such as tobacco smoking abstinence either is or is not enacted depending on interactions between factors across three components: *capability* (physical and psychological), *opportunity* (social and physical environment) and *motivation* (reflective and autonomic) [62, 63]. We coded the entire dataset against the COM-B model in NVivo20. Sub-themes related to components in the COM-B model (e.g. capabilities, opportunities, motivations) were developed (by AB) and refined over multiple iterations, informed by the Theoretical Domains Framework [64] and discussions within the research team. It was common for data to potentially map to multiple areas in the COM-B model; decisions about how to present data were informed by close reading of extracts, and pragmatic considerations to reduce unnecessary repetition. Our aim in mapping the interview data against the COM-B model was to try to develop a rich and multi-faceted understanding of potential enablers and barriers to post-release tobacco smoking abstinence; and inform thinking about what strategies might be successful in reducing post-release smoking relapse following release from smoke-free prisons in the future. Themes were derived from a combination of participants’ expectations of enablers or barriers to achieving smoking abstinence after leaving prison and, in some cases, their actual experiences of trying to stop or stopping smoking in the recent past. Themes are based on our own interpretation of the implicit meaning of data as well as issues explicitly discussed by participants. Extracts from interviews are attributed to participants (person in prison, ‘PiP’) using a serial number; for staff it includes a very broad description of their job role (prison or other) for confidentiality reasons.

## Results

Potential barriers and enablers to smoking abstinence after leaving a smokefree prison where vaping is allowed are presented below in relation to the components of the COM-B model of behaviour change.

### Capability

#### Beliefs about smoking harms

There was little in the interviews to suggest that lack of knowledge about the harms of smoking was a major barrier to being tobacco-free post-release. However, people in prison often rationalised smoking in the face of seemingly compelling health and/or money-related reasons for stopping (PiP01-03: *£15 [for a pack of cigarettes]*,* that’s alright…It is what it is”*), and this was identified as a potential barrier. Smoking rationalisations included references to the pleasure or enjoyment that comes from smoking, the perceived value of smoking for managing stress, and associating smoking with regaining control and autonomy post-release. PIP02-06 suggested that it was better to live a shorter life than to forgo the ‘benefits’ of smoking.

*PiP02-06: “I will probably…continue to smoke and vape [post-release]. I know it is crazy to say this…nobody lives forever anyway. I am a mad one*,* not a sad one.”*

#### Self-efficacy

Holding low expectations (PiP02-01: *‘it is what it is’*) about sustaining smoking abstinence post-release was identified as a potential barrier, influenced by factors such as previous lack of success in maintaining abstinence after leaving prison or lack of success with other quit attempts. Perceptions that being tobacco-free would be very challenging or even unattainable in the context of anticipated personal and environmental triggers post-release undermined confidence in quitting:

*PiP02-02: “I’ve been in this position before and the minute I walked out the gates [of the prison after being released]*,* the first thing I did is [go] to the shop and [buy] 20 [cigarettes]. So I think this is what will happen this time [I’m released] as well.”*

*PiP02-01: I’m actually hoping to stay smokefree. I mean*,* see to be honest*,* I know that when I drink and I have a can [of beer]*,* I will [smoke] a wee bit.*

Holding seemingly unrealistic expectations about life post-release either in general or specifically in relation to successfully staying tobacco-free was also identified as a potential barrier. For example, PiP02-03 asserted he was “100%” confident he would manage to remain abstinent post-release because he believed he could avoid stressors which had caused him to relapse in the past. In contrast, holding more realistic expectations of success was identified as potential facilitator, as illustrated by PiP01-15 who said he had come to realise the importance of planning for being tobacco-free after release to avoid repeating previous patterns of behaviour:

*PiP01-15: “I’ve been in and out of prison all my life*,* and every time I’m going out*,* I say*,*…I’m not going to do this [smoke tobacco again]…and I just do it anyway.”*

### Opportunity

#### Vaping

Vaping was identified both as a potential barrier and facilitator to people maintaining smoking abstinence post-release, depending on the circumstances. In respect to barriers, PiP01-08 believed that one of the reasons he had returned to smoking after leaving prison on a previous occasion was because he had sustained his nicotine dependence during his prison stay through vaping, which had fuelled his cravings for cigarettes: *“I had to go and buy 20 [cigarettes] at the shop [immediately after leaving prison]…The vaping just doesn’t change it. It just makes you want [tobacco] more…”* Similarly, PiP01-13 expected that, despite his interest in cessation, he would likely return to smoking post-release because he felt that, by vaping in prison, he had not *“gave smoking up altogether*,* which would be a good thing*”. Strong preferences for smoking over vaping or ambivalence towards vaping was a key reason why some people expected to switch from vaping whilst in prison, where smoking was not a choice they could make, back to smoking post-release. Low satisfaction/enjoyment of vaping was cited as a reason for negative or ambivalent perceptions of vaping:

*Staff-01 [Prison]: “smoking is…the preferred method of getting your nicotine*,* and [some people in prison] just make do with a vape when [they’re] in here.”*

*PiP01-15: “I’m considering [being smokefree post-release]*,* aye…but having a choice is hard…because a vape doesn’t really do it…it’s not [tobacco]”*.

Another key reason for negative or ambivalent attitudes to vaping related to reported negative side effects from vaping in prison and concerns about health harms: *“I keep on trying to stop them [vapes] because I know they are doing damage to my lungs”* (PiP02-06). Negative or conflicting attitudes and experiences of vaping, and desires to withdraw from nicotine to assist with being tobacco-free post-release, were key motivators for some people wishing to quit (or cut down) on vaping prior to release.

In contrast, PiP01-06 said he had switched to vaping in prison on a previous occasion and then continued to vape exclusively post-release: *“I was vaping when I got out…last year. I was vaping right up to going to jail again.”* Staff-04 [other] confirmed “…*[some people tell me] ‘I…didn’t go back to smoking [post-release]*,* stayed vaping’…it’s not huge numbers [of people]*,* but again I’m not seeing a huge amount of the population.”* PiP01-07 suggested that vaping could potentially help some people to avoid returning to smoking post release because vaping was seen “*as an extension of smoking*”, meaning that “*there was no [need] to stop*”, but rather to switch from one product to another. Another suggested reason that vaping may help support post-release smoking abstinence was that vaping can be a way for people to reduce dependence on nicotine in prison, as illustrated by PiP02-02: “*I was seriously considering stopping smoking when I get out because…if I…I’ve not got money for the pods [e-liquids] just now*,* I’m not climbing walls and that*,* like I usually [would] do if it was tobacco.”* Vaping instead of smoking outside of prison was appealing for some on health and financial grounds and because it provided an alternative way for trying to manage negative emotions, adverse circumstances and stressors.

#### Social influence

Negative social influence related to prevailing social norms around smoking among the social networks people were returning to after leaving prison, was identified as a potential barrier. Interviews reflected very high rates of smoking (PiP02-11: “*all my family…smoke*”) and greater social acceptance of smoking (PiP02-01: “*it’s the way you’ve been brought up*”) within the communities that many people in prison were returning to. Smoking (and, in some cases, use of other substances) was portrayed as an integral part of “*normal”* life (PiP01-10) outside prison and a shared activity within social networks. PiP02-01 explained that despite withdrawing from nicotine in prison on a previous occasion, he had quickly resumed using substances post-release due to his return to “*hanging out*” with “*folk*” for whom smoking and drinking was part of day-to-day life. Acceptance of smoking among social networks meant that some people like PiP01-10 were given *“a carry-out [alcohol] and fags [cigarettes]”* by the person who met them at the prison gate. Successfully staying tobacco free in the face of being surrounded by people smoking within living and social environments was generally recognised as challenging:

*PiP01-15“…you’ve got all good intentions of*,* ‘right*,* I’m not going to smoke*,* I’ll go and get a pint’. Go and have a pint*,* go to walk out and you see somebody with [tobacco]*,* the first person you see you ask*,* ‘You got [tobacco] on you?’ Right back on it…it’s hard.”*

*Staff-02 [prison]: a lot of guys might be fine Monday to Friday*,* and Saturday night comes…their pals [friends] who have been working*,* they do kind of manage their drink or their drugs*,* they go and meet them and they might go off the rails.*

*PiP02-09: “that smell of smoke. That could be a trigger…my mum and dad*,* they smoke*,* so if I was going…to visit them…it would give you an urge to smoke.”*

Being placed in hostel accommodation after leaving prison was noted to leave people particularly vulnerable to this negative social influence in relation to tobacco and substance use relapse:

*PiP01-15: “I’ve struggled with drugs [before coming to prison*,* but] I’ve been clean for nine months now. If I go back into a hostel*,* I’m going to use again*,* and nobody seems to give a toss [care]…”*.

In contrast, positive social influence was identified as a potential facilitator. For example, PiP02-05, who wanted to stay free of tobacco, alcohol and illegal drugs after leaving prison, believed that achieving his goals would be “*a lot easier*” than previously as he would be returning to a supportive family environment this time rather than to “*people wanting me to go and get drunk*”. He went on to explain “*my partner doesn’t smoke*,* and I’m not allowed to smoke in the house”*, which may support efforts to be tobacco-free (or to smoke at reduced levels in the event of a relapse). On a larger scale, positive social influence due to national declines in smoking rates and the introduction of smokefree public spaces were also identified as potential facilitators:

*PiP02-04…the older I’ve got*,* the less people I know to smoke now. Even the younger generation*,* a lot of people don’t smoke any more…*.

***Interviewer: do you find you really notice it, with going in and out?***

*PiP02-04: Yeah*,* I do notice it*,* yeah. And I think again with the pubs*,* I think with the government putting the*,* kind of*,* ban on smoking in public has had a huge effect.*

#### Tobacco availability

Tobacco products being widely available to purchase, particularly in socially disadvantaged neighbourhoods was identified as a potential barrier. This was reflected in people’s descriptions of buying tobacco (and other products which are not permitted in prisons, such as alcohol) with ease almost immediately after being released from prison on a previous occasion.

*PiP01-02: every time I’ve gone out, I’ve ended up smoking again*….

***Interviewer: So***,*** why do you think that is***….

*PiP01-02: you’ve got it in your head*,* ‘No*,* I’m not going to do that’…and then you get out*,* and the shop’s just down the bottom of the road.*

#### Support services

As noted, in Scotland, all NHS Boards are required to comply with the minimum requirements for NHS cessation (‘Quit Your Way’) services in prisons [48]. The offer of cessation support in prisons was identified as a potential facilitator. Several challenges related to offering cessation services in prisons were also described in interviews, and, conversely, these were identified as barriers. Greater difficulties delivering essential health and rehabilitative services and ensuring the safe operation of prisons has been identified as one of the many harms caused by prisons caring for a rising and ‘more diverse’ [50] prison population, and the legacy of the COVID-19 pandemic in prisons [65–68]. This was reflected in the interviews. Changes to the prison regime necessitated by managing the COVID-19 pandemic within prisons had severely restricted health and other services in the period immediately preceding the interviews [68], resulting in gaps in provision and “*huge waiting lists*” (Staff-04 [other]) for cessation support (and more broadly) when services reopened. While staff were working hard to reduce waiting times, these were often considerably longer than desired due to reduced service capacity and efficiency compared to the pre-pandemic period, which could result in some people becoming de-motivated to address nicotine dependence:

PiP01-11: *No reply back [after submitting a referral form for the cessation service]. Then I…thought*,* ‘Oh*,* what’s the point? I’m trying*,* and you just get a blank.’*

Practical issues, such as difficulties accessing suitable spaces and having to work to more constrained prison regimes, reduced the reach of cessation services at that time. Staffing levels, logistical challenges and a high prison population meant cessation services could not “*do everything that we want to do*” (Staff-03 [other]), including being able to more widely support people coming up for release who were motivated to remain tobacco-free. As prison leaders and staff were facing substantial competing priorities and pressures in the context of Scotland’s growing, overcrowded and increasingly complex prison population [67], it is understandable that the prison service was perceived by Staff-03 [other] to be less able to fully support cessation services, post implementation of smokefree prisons, especially in the immediate aftermath of the COVID-19 pandemic. This was identified as a barrier, although Staff03 [other] noted that overall *“[prison] teams and managers…support our [cessation] services very well”.*

The interviews also reflected the variability in throughcare support in general for people leaving prison, particularly those on short-term sentences. Interviews confirmed that inadequate support in the period leading up to and after release can make it extremely challenging for people to transition from prison to community successfully and can result in people having a plethora of unmet needs which overlap with known risk factors for smoking. Further, Staff-08 [other] suggested that getting timely access to cessation support in the community was likely to be challenging in the current fiscal environment: Staff-08 [other]: *“funding is not sufficient to provide the service that we need”* and “*we are continually asked to do more with less staff and less funding.”*

### Motivation

#### Motives for smoking or abstaining

As noted above, people offered rationalisations for wanting to smoke given their life experience, and some referred to their enjoyment of smoking. These stated reasons for smoking were identified as a potential barrier to remaining tobacco-free:

PiP02-02: *I do enjoy it [smoking]…even when my mum was told she had cancer [she] just couldn’t do it [stop smoking]. She enjoyed it too much she said. And I think…I don’t know if it’s because I used to hear my mum saying that for years and years*,* ‘Oh I enjoy smoking*,* I couldn’t give up!’…it’s the only thing she’s got…*.

In contrast, having positive reasons for being tobacco-free was a potential facilitator. The rising price of tobacco (outside prison) was discussed as a major incentive for remaining smokefree: PiP-04: *I’d never go back to smoking cigarettes again…there’s no point going back. I’ll save a fortune.”* Wanting to improve health, sustain perceived improvements in health while living in a smokefree environment (PiP02-09 “*I feel like I’m more energetic*”), or prevent developing smoking-related diseases in later life (PiP01-11: *I’ve got to stop it now. Before I end up with chronic health problems and breathing problems.”)* were other motivators. Wanting to protect and show consideration for the health of others could be another important motivator, as illustrated by PiP01-16 who had a close family member with advanced cancer who had themselves recently quit smoking. Other motivators to remain smokefree included dislike of the taste and smell of tobacco and a perception that smoking cannabis (with tobacco) was a barrier to achieving other goals post-release, such as finding suitable employment. Wanting to break free of addiction to smoking tobacco alongside other substances was a strong motivator for PiP01-01 who said he had been in a *“lifelong battle*” with his “*nemesis*,* addiction*”.

#### Interest in smoking cessation

A recurrent theme in the data was interest in smoking cessation, which was identified as a facilitator: PiP01-01: “*I think everybody that smokes wants to stop”*. Some participants expressed complex feelings about quitting, reflecting tensions between the perceived benefits of smoking vs not smoking:

*PiP01-12: I’ll go months [without smoking cannabis cigarettes*,* outside prison] and there’s just something about it*,* I’m drawn back to…I think that’s what the problem is*,* I’ve seen it as being acceptable…but then…my kids…doing it…would bother me…So*,* why is it alright for me to do it? I don’t have the answer for that.*

Being *“forced”* to go without tobacco in prison fuelled some people’s interest and motivation to continue abstinence post-release. However, Staff-04 [other] recognised there were also people in prison who had strong interest in returning to smoking *“as soon as I get out of here”*, which was identified as a barrier.

#### Prioritising smoking abstinence alongside multiple health and social needs

People in prison often have multiple and complex needs which, although often interlinked with smoking, may be a much higher or more immediate priority for them. This was reflected in the interview data. Challenges that people discussed included mental health problems, often related to adverse circumstances in childhood, past trauma, long-standing addiction to alcohol and/or illegal drugs, lack of suitable accommodation post-release, relationship breakdowns and financial and employment difficulties.

*Staff-06 [prison]: “…most of the chaps we tend to get have experienced a lot of trauma…some of the stories are quite harrowing.”*

*PiP02-07 “Scotland must be up there in the top ranking [countries] for drug [related harms]…that’s where a lot of people’s [in prisons] stuff [problems] stem from is through drug taking*,* through stealing [to fund their]… habit…”*.

*PiP02-10: I was outside [prison] not that long ago and when I went out [was released] I felt overwhelmed. It was difficult. I didn’t have any phone to phone the Job Centre*,* to do the online benefits system. I was left in the streets with no money*,* no place to go*,* no house and no family*.

Remaining smokefree was understandably described as being of lower priority in the face of such immediate health and social problems, and this was identified as a potential barrier. PiP01-07, who had experience of mentoring others in prison, said that people rarely mentioned smoking when preparing for release because smoking was “*probably last on their list*” of problems to worry about. Similarly, PiP02-02 said *“I’ve got too many other things…bigger problems to think about than the smoking”.* In the context of multiple needs, interviews with both staff and people in prison suggested that the issues (e.g. alcohol and illegal drug use) which were perceived to place people at greatest risk of reoffending and caused significant problems in day-to-day life were often prioritised: *“I can’t [go back to drinking]…because when I drink*,* it leads to all drugs*,* and all drugs leads to chaos*,* and chaos leads to jail”* (PiP01-15). By contrast, continued smoking was something some people felt able to “*cope with*” (PiP01-01).

Smoking cessation was also sometimes seen as having lower salience than competing health and social needs, among the support networks of people in prison. Staff-05 [other] commented that smoking cessation was likely *“very low down the list of things….to…worry about”* for some families affected by imprisonment, who were often focusing on supporting someone leaving prison with more immediate challenges such *as “trying to get food for that person*,* or their medication sorted…. not knowing about housing*,* all these things…”*. While benefits for individuals and for the children of parents who quit smoking (or made their home smokefree) were recognised by Staff-06 [prison], they expressed uncertainty about the relative importance of smoking cessation support in prisons given practical challenges for delivering services and competing issues:

*Staff-06 [prison]: “is there really a need to address [smoking]…how high is smoking? Do people view it as quite a bad addiction and does it need treated?…you’d really need to figure out is it going to be beneficial to people to go on a journey with their non-smoking.”*

Staff-08 [other]’s perception based on their experience of supporting people in disadvantaged communities was that, in relation to supporting cessation, *“everybody [outside cessation services] thinks that’s somebody else’s job”.* Staff-08 [other] perceived that, despite the substantial burden of smoking on society, the relative importance of tobacco as a public health issue could sometimes be seen as a lower priority than other substance dependencies and was further deprioritised during the COVID-19 pandemic:

*“…[around] 9000 deaths [in Scotland] are caused as a result of smoking…that’s a huge number…The number of deaths caused by drugs*,* we’re sitting at 1300. I think we’re sitting at 1400 for alcohol deaths. I don’t understand why tobacco is not being prioritised…Why are people not shouting about the drug deaths caused by cigarettes?…and the wider cost implications on the [NHS due to the] number of hospital admissions caused by smoking. [S]moking doesn’t happen in a vacuum…when they’re drinking*,* they’re smoking*,* and more likely to engage in riskier behaviour such as taking drugs.*

#### Tobacco, alcohol and drugs

Polysubstance use was identified as a substantial potential barrier. Habitual co-use of tobacco and cannabis (e.g. cannabis cigarettes) prior to imprisonment was frequently reported: PiP 01–15 “*I don’t smoke tobacco on its own…I would use [tobacco] with cannabis”.* Intentions to revert to smoking cannabis with tobacco post-release were justified in relation to enjoyment, perceptions that cannabis use was socially *“acceptable”* (PiP 01–12), and beliefs that smoking cannabis was a less harmful alternative to ‘*harder*’ drugs: PiP01-12, “*there are a lot worse things I could be doing”.* Established rituals and associations between alcohol, illegal drugs and tobacco smoking, and alcohol and drug use being drivers of more intense smoking behaviour were identified as a challenge for smoking cessation.

*PiP01-11: “As soon as I have a can of…beer or whatever… I want a [cigarette]. On nights out and that…when I’m out [of prison]*,* I do smoke*,* but it’s only when I’m really drinking*,* or cannabis…It’s mostly joints [I smoke]*,* but that’s still tobacco…*.

*PiP01-04: I used to…[take] cocaine…And [when I did] I used to smoke non-stop*,* like a chain*,* it was non-stop…”*.

#### Addiction

Ingrained habits and rituals associated with smoking outside of prison were seen as potential barriers. For example, PiP01-16 described how smoking cannabis cigarettes and drinking a “*cup of tea*” went “*hand in hand*” in his life prior to imprisonment. PiP01-08 described smoking as an “*unconscious*” behaviour which he had found difficult to quit in the past. Several participants described smoking as an “*addiction”* and reflected on difficulties they or other people had faced prior to imprisonment in resisting or satisfying strong cravings:

*PiP02-10 “I have smoked since I [was] young*,* so it is an addiction…always when I get money I go to the shop and get tobacco…and then go back home. Then you are smoking a pouch of tobacco like it is going out of fashion. It is madness the amount we smoke.”*

In contrast, decreases in cravings and urges to smoke whilst living in a smokefree environment, and forming a “non-smoker” identity, whilst in prison were identified as potential facilitators.

*PiP02-07: “I’ve took a lot of positives out of prison… now*,* I’m a non-smoker and I have been for four years.”*

#### Negative emotions

Negative emotions such as stress, anxiety and boredom were recognised as common barriers to smoking cessation, alongside strong personal and cultural associations of smoking with comfort or relief of stress. There were suggestions that returning to smoking was almost inevitable given that smoking was an ingrained response to individual-level, interpersonal and environmental stressors, which were common post-release:

*PiP01-08: “…when you get out [of prison]*,* you’ve got nothing else to do apart from stress…cause there’s nothing for us out there.”*

## Discussion

Consistent with the limited evidence from other countries [33, 34, 43], interviews identified substantial potential barriers to increasing smoking abstinence among people leaving smokefree prisons. Interactions between people’s rationalisations for returning to tobacco smoking post-release (‘capability’), tobacco availability (post-release), smoking norms, service limitations (‘opportunity’), competing needs and priorities and drug and alcohol use (‘motivation’) were identified as potential barriers. Overwhelmingly, variability in support in transitioning back into the community meant that a desire to remain tobacco-free was often dwarfed by multiple immediate challenges, such as applying for benefits or jobs, or finding somewhere to live. Opportunities for increasing smoking abstinence rates among people leaving smokefree prisons were also identified. Many participants expressed desires to quit smoking for good given recognised health harms, rising tobacco prices and desires to break free of ‘addictions’ which have caused substantial damage in their own and others’ lives. Ongoing delivery of ‘smoking cessation’ services in Scottish prisons post-implementation of smokefree policies was identified as a potentially significant opportunity for reducing tobacco harms among people leaving prison. Opportunities to capitalise on and strengthen bonds between people in prison and those in their networks who can encourage and support positive behaviour change were also identified.

Our study adds to existing evidence in being, to our knowledge, the first to study potential barriers and enablers to remaining abstinent from smoking after release from smokefree prisons where vaping is permitted. Our novel findings suggest there are both perceived risks and opportunities for post-release smoking abstinence among people leaving smokefree prisons where they have had access to vapes. As in the general population [69], our data suggest that switching from tobacco to vaping in prison could help some people to remain smokefree post-release by, for example, enabling them to find an acceptable or preferable alternative to smoking and/or helping to reduce nicotine dependence prior to release. However, vaping is unlikely to help with post-release smoking abstinence among people with low satisfaction/enjoyment of vaping or concerns about potential health harms. Maintaining a degree of nicotine dependence and/or similar habits/rituals to smoking by vaping in prison could also lead some to struggle with post-release smoking abstinence, especially as evidence suggest that those vaping in prison may vape intensively/in ways which may not be optimal for cessation. Vaping practices in prison, including vapes being a common route to administer illicit drugs [57], may mean that the risks/benefits to individual and population health from vaping may differ between prison and community.

Consistent with our earlier studies conducted with people in prison in Scotland [58, 70], interest in reducing or stopping vaping prior to release was often expressed in interviews, for reasons such as dislike or perceived harms of vaping or to support long-term smoking abstinence. Taken together, findings highlight the value of a person-led approach to reducing tobacco-related harms in prisons which may involve supporting some people to vape for smoking cessation, while helping others who wish to reduce or stop vaping. Offering information and support to people who vape in prison could provide additional opportunities to increase motivation to stay tobacco-free on release, give people who are dissatisfied with vaping access to support and alternative strategies to remain tobacco-free long-term, or support and provide confidence and reassurance to those seeking harm reduction options.

Renewed efforts to tackle smoking inequalities, e.g. through support for smoking abstinence for people leaving smokefree settings such as prisons and those living in more disadvantaged communities, is essential if societies are to achieve ‘tobacco-free’ generation targets as have been set in several (largely high-income) countries [43]. Given the interconnections between smoking, use of other substances, and poor mental health, improving and tailoring smoking cessation support for people with experience of imprisonment would likely also benefit work in other critical areas of public health and social justice [44]. Further, increasing the number of children with parents who do not smoke or who live in smokefree homes is beneficial for child health, and can help to break intergenerational cycles of smoking, which are now concentrated in the most disadvantaged communities, and so reduce health inequalities [45]. While findings indicate there is substantial potential to help people to overcome or better manage nicotine dependence in prison and to support long-term abstinence post-release, greater success depends on finding ways of working within, or reducing, key constraints.

For example, our findings highlight how challenging it is for people leaving prisons to stay smokefree in the face of multiple, often pressing and complex health and social needs. Delivering services which identify, and address people’s needs more holistically may be essential to increasing post-release smoking abstinence, as well as reducing some of the underlying social determinants of poor health and involvement in crime. While the value and acceptability of addressing smoking concurrently with other social and health needs has been demonstrated in community health settings [71, 72], it is unclear whether and how a more holistic and person-centred approach to smoking cessation could be realised in prisons. Key challenges include substantial health and prison service resource limitations in the context of growing and increasingly complex need within the prison population [67], structural barriers to collaborative working within and between health and justice organisations [66] and the substantial health risks posed by smoking not being adequately reflected in substance use strategy and service delivery in Scottish prisons, as indicated by greater focus on alcohol and drugs than on tobacco. Strengthening pathways within and between prison and community services addressing different social, health and criminogenic needs may be a more achievable strategy for supporting people to make the multiple, interconnected, behaviour changes that many people in prison say that want to make. Positive changes in one area can increase willingness, confidence and capacity to make other positive lifestyle changes [73]. The launch in 2025 of a new voluntary prison throughcare service in Scotland which offers people leaving prison, after serving a short-term sentence or a period on remand, reintegration support and help to access wider services could provide new opportunities to implement evidence-based strategies for smoking cessation among prison leavers. For example, brief advice and referral interventions may be particularly effective when delivered in a sensitive and person-centred way by support workers who have built trusting relationships with individuals and understand their broader priorities and goals [74]. However, as in other settings [75], constraints such as time pressures, competing priorities and staff lack of knowledge may be obstacles to integrating tobacco use interventions in prison throughcare services.

Peer support models are another way of potentially supporting people in prisons holistically to address smoking and other related health and social needs. Peer delivered programmes in prisons can deliver benefits across a range of outcomes [76], including for smoking cessation [40]. However, peer supporters in prisons can experience significant challenges related to the physically and emotionally demanding nature of their volunteering, inconsistences in training and support, and managing high levels of unmet need resulting from pressures on core services [77]. Scaling up peer support models in prisons, underpinned by appropriate training and supervision, to support people to stay smokefree post-release and make and sustain other health behaviour changes requires careful consideration in the current penal climate. Use of new technology in prisons during the COVID-19 pandemic may create greater opportunities for digital health interventions, including providing information on vaping to help address misperceptions and erroneous or illicit use [78]. Whichever way services may develop in the future it will be important for services to keep pace with the rapidly changing tobacco and nicotine product landscape outside of prisons.

Another significant barrier to more effectively reducing tobacco-related harms among people leaving prison is the limited robust evidence on strategies which may help people to stop vaping without comprising smoking abstinence [79] and on what helps people to remain tobacco-free after leaving smokefree settings [32, 33]. Findings also confirm the importance of further research to better understand feasible and effective approaches for reducing harms among people who use tobacco with other substances and/or who use vapes to administer illicit drugs in prison.

A major strength of this study is that it provides evidence from the perspectives of people imprisoned and staff working in prisons which are smokefree but allow vaping. We were able to deliver the study between 2021 and 2023, albeit with some modifications, during a challenging climate for prisons and prisons-based research exacerbated by the aftermath of the COVID-19 pandemic [78], and an increasing and more complex prison population. A combination of these factors disrupted the study, resulting in unavoidable but substantial delays to data collection, and changes to the study protocol to ease the burden on overstretched prison and health services. Some of the difficulties we encountered in conducting the study may reflect the challenges of keeping smoking and vaping high on the agenda in prisons in the face of so many competing priorities and pressures. Although we conducted fewer interviews with people in prison than planned, we believe we were approaching data saturation by the end of fieldwork, suggesting there would have been minimal gains in conducting more interviews, particularly given the need to minimise the impact of research demands on the prison and health services. Further informal discussion with additional people in prison, during numerous visits, reinforced our findings from the interviews, rather than highlighting any important insights we had missed. Nonetheless, our study comprises a self-selected sample and it is possible that the views and experiences of those who chose to participate may not fully reflect the diversity of the prison population. Had circumstances been different we intended to conduct some follow-up interviews with people released from smokefree prisons to explore smoking behaviour. Loss to follow up has been noted as a particular challenge in prison research [80], and problems were compounded by delays and resource constraints. An additional limitation is that some key perspectives may be missing from the staff interviews because of recruitment difficulties due to current staffing pressures.

## Electronic supplementary material

Below is the link to the electronic supplementary material.

Supplementary Material 1

## Data Availability

The qualitative datasets generated during the current study are not publicly available due to difficulties sufficiently de-identifying data to protect research participants, and lack of consent from participants for data sharing. Please contact the corresponding author to discuss access requests.
