# Supplementary material for: Identifying potential barriers and enablers to smoking abstinence after leaving a smokefree prison using the capabilities, opportunities, motivations -behaviour (COM-B) model: a qualitative interview study
Source: BMC Public Health. 2025 Jun 5;25:2100. doi: 10.1186/s12889-025-23249-3 (PMC12139055; doi:10.1186/s12889-025-23249-3)
Supplement: Supplementary file 1 — Supplementary Material 1 [file 12889_2025_23249_MOESM1_ESM.docx]

**Barriers and enables to post-release smoking abstinence**

**High level summary of topics covered in interviews with staff and people in prison**

Topics covered in interviews with people in prison:

- Participant background/ wider context
- Smoking and vaping history
- Living in a smokefree prison
- Preparing to leave a smokefree prison
- What might work to help people leaving prison and families who want to be smokefree or make their homes smokefree.

Topics covered in interviews with staff:

- Participant background
- Challenges and opportunities for work on smoking/vaping in prisons/wider society
- Smokefree prison policies and supporting people in prison to manage without tobacco
- Vaping in prisons
- Release from smokefree prisons, including opportunities and challenges for reducing tobacco-related harms.
